# Inhibition of the long non-coding RNA *NEAT1* protects cardiomyocytes from hypoxia in vitro via decreased pri-miRNA processing

**DOI:** 10.1038/s41419-020-02854-7

**Published:** 2020-08-13

**Authors:** Olof Gidlöf, Kerstin Bader, Selvi Celik, Mario Grossi, Shinichi Nakagawa, Tetsuro Hirose, Bernhard Metzler, Björn Olde, David Erlinge

**Affiliations:** 1 Department of Cardiology, Clinical Sciences, Lund University, Lund, Sweden; 2 University Clinic for Internal Medicine III, Cardiology and Angiology, Medical University of Innsbruck, Innsbruck, Austria; 3 Institute for Genetic Medicine, Hokkaido University, Sapporo, Japan

**Keywords:** Apoptosis, Long non-coding RNAs, miRNAs, Myocardial infarction

## Abstract

While restoration of coronary blood flow to the ischemic heart is the most effective strategy for reducing infarct size, reperfusion injury represents a significant limiting factor on clinical outcomes in myocardial infarction patients. Ischemic preconditioning (IPC) has been shown to inhibit reperfusion injury and represents an attractive model for studying cardioprotective signal transduction pathways. Long non-coding RNAs (lncRNAs) are a structurally and functionally heterogenous class of RNA transcripts with unknown roles in IPC-induced cardioprotection. Through microarray-based expression profiling of 31,423 lncRNAs in cardiac tissue from IPC mice, we identified the nuclear transcript *Neat1* to be rapidly and robustly decreased in response to IPC. siRNA-mediated knock down of *Neat1* reduced apoptosis and necrosis in murine cardiomyocytes (CM) and human iPS-derived CMs in response to prolonged hypoxia and hypoxia-reoxygenation, assessed with Annexin V/propidium iodide-staining, a Caspase 3/7 activity assay, LDH release, and western blot for cleaved Caspase 3. Mechanistically, Neat1 was shown to regulate processing of pro-apoptotic microRNA-22 (miR-22) in murine and human CM nuclei using a luciferase reporter assay. Hypoxia-induced downregulation of Neat1 was shown to result in accumulation of unprocessed pri-miRNA and decreased availability of biologically active miRNA, including miR-22. Addition of exogenous synthetic miR-22 reversed the protective effect of *Neat1* knock down in human iPS-CM. In conclusion, we have identified the nuclear lncRNA *Neat1* as part of a conserved oxygen-sensitive feedback mechanism by regulation of miRNA processing and a potential target in cardioprotection.

## Introduction

Restoration of coronary circulation is vital in limiting ischemic injury and improving outcomes after myocardial infarction (MI), but the act of reperfusion does in itself cause irreversible injury to the myocardium^1^. While improved prevention and treatment strategies have reduced mortality in MI^2^, an increasing number of patients are suffering from the consequences of ischemia/reperfusion, i.e. progressive myocardial remodeling and heart failure^3^. Thus, therapeutic strategies to protect the myocardium after reperfusion is an unmet clinical need. Ischemic preconditioning (IPC), i.e., short repeated episodes of non-lethal ischemia, has been shown to substantially reduce infarct size and improve cardiac function in various animal models of MI^4^. While the molecular underpinnings of IPC-induced cardioprotection are still incompletely understood, and the fact that the treatment must be performed prior to the ischemic insult means that it will never be clinically useful, IPC represents an attractive model to identify and study cardioprotective pathways.

IPC confers protection during two distinct temporal phases, an early phase which begins immediately after treatment and lasts for 2–3 h, and a late phase which begins 12–24 h after treatment and lasts 48–72 h^5^. While the first window of protection is thought to be dependent on acutely available signaling molecules, the second window is likely to involve activation of specific gene expression programs and de novo synthesis of protective proteins. IPC has been shown to trigger a diverse array of transcriptional activators, including NFKB^6^, STAT1, and STAT3^7,8^, and while the protein-coding transcriptome has been shown to be profoundly perturbed in the response to the treatment^9,10^, no study to date has comprehensively examined the effect of IPC on the cardiac non-coding transcriptome. Non-coding transcription is pervasive throughout the mammalian genome^11^, giving rise to >100,000 different unique transcripts^12^. For an overwhelming majority of these transcripts, a biological role has yet to be established. In the context of cardiac IPC, a number of studies points to distinct roles for the microRNA (miRNA) class of non-coding RNAs. Yin et al reported that miRNAs play a role in activating endothelial nitric oxide synthase and the heat shock pathway in late phase IPC cardioprotection^13^ whereas Cheng et al. showed that miR-21 is triggered by IPC and confers a cardioprotective effect through repression of the *PDCD4* gene^14^. The long non-coding RNA (lncRNA) class comprise a group of structurally and functionally diverse transcripts with known roles in cardiac development^15,16^ and disease^17,18^ but evidence of a mechanistic link between cardiac IPC and lncRNA is still elusive.

The aim of this study was to map and mechanistically characterize the cardiac lncRNA transcriptome in response to IPC to identify novel cardioprotective pathways.

## Methods

### Mouse model of IPC

All procedures were performed according to protocols approved by the Institutional Committee for Use and Care of Laboratory Animals, University of Innsbruck. Mycoardial ischemia-reperfusion in mice was induced as previously described^19^. In short, male and female C57BL6 mice were anesthetized by intraperitoneal injection of medetomidine (0.5 mg/kg), midazolam (5 mg/kg), and fentanyl (0.05 mg/kg). Ischemia/reperfusion was achieved by sequential tightening and loosening of a silk suture around the left descending coronary artery. Mice were subjected to four cycles of 5-min ischemia and 5-min reperfusion, followed by 30 min of reperfusion. Myocardial tissue was harvested and perfused with PBS and biopsies from the ischemic area (area at risk, AAR) in the immediate proximity of the ligation, and nonischemic area (remote myocardium, RM), excised from the right ventricle, were collected and stored at −80 °C. For RNA isolation, myocardial biopsies were placed in QIAzol and homogenized using an Omni TH rotor-stator homogenized (Omni International, NW Kennesaw, GA, USA). Total RNA was prepared using the miRNeasy mini kit (Qiagen, Hilden, Germany) according to the manufacturer’s instructions and stored at −80 °C.

### Cell culture

HL-1 cells were kindly provided by Professor William C. Claycomb, Louisiana State University Medical Center, New Orleans, USA. Cells were grown in Claycomb medium (Sigma Aldrich, St. Louis, MO, USA) supplemented with 10% FBS, 100 U/mL of Penicillin/Streptomycin, 0.1 mM Norepinephrine, and 2 mM l-Glutamine and 25 mM HEPES. Culture flasks and plates were coated with 0.02% gelatin and 5 μg/ml fibronectin for 1 h before seeding cells.

Rat neonatal cardiomyocytes were acquired from Lonza (Basel, Switzerland) and maintained in Rat Cardiomyocyte Growth Medium according to the manufacturer’s instructions.

Human iPS-derived cardiomyocytes (iPS-CM) were bought from Cellular Dynamics International, Madison, WI, USA. Cells were thawed and grown in Plating or Maintenance Medium according to the manufacturer’s instructions. All treatments were carried out at day 5 post plating.

In all cells and cell lines, absence of mycoplasma infection was guaranteed by the providers.

For hypoxia experiments, cells were placed in a sealed chamber within a regular cell culture incubator and connected to a ProOx 110 O_2_ controller (Biospherix, Parish, NY, USA). pO_2_ was kept at 1% by continuous infusion of N_2_. 24 h prior to hypoxia, cell medium was changed to hypoxia assay medium (DMEM w/o glucose, glutamine, and phenol red, supplemented with 5 mM Creatine, 2.75 mM d-(+) Glucose, 1X GlutaMax, 10 mM HEPES, 2 mM l-Carnitine, 1X Sodium Pyruvate, 5 mM Taurine, and 1X Linoelic-oleic acid). Cells were transfected with siRNA, pre- and anti-miRNA and plasmid DNA using Lipofectamine RNAiMax or Lipofectamine 3000 (Thermo Fisher Scientific, Waltham, MA, USA) according to the manufacturer’s instructions. siRNA for human and mouse *NEAT1* and *SFPQ* or scrambled negative control siRNA (Thermo Fisher Scientific) were transfected at a concentration of 40 nM. Pre- and anti-miR-22 or respective negative controls (Thermo Fisher Scientific) were transfected at a concentration of 40 nM. A total of 100 ng of plasmid DNA was used per transfection. Total RNA was prepared from cells using the miRNeasy mini kit (Qiagen) according to the manufacturer’s instructions and stored at −80 °C.

### lncRNA array

Expression profiling of 31,423 long non-coding RNAs was performed on AAR and RM biopsies from IPC mice (*n* = 2 per group) by ArrayStar Inc. (Rockville, MD, USA) using the Arraystar Mouse LncRNA Array v 2.0. Quality and integrity of the samples were determined using NanoDrop ND-1000 and denaturing agarose gel electrophoresis. Sample labeling and array hybridization were performed according to the Agilent One-Color Microarray-Based Gene Expression Analysis Protocol (Agilent Technologies, Santa Clara, CA, USA). Labeled cRNAs were purified by RNeasy mini kit (Qiagen). The concentration and specific activity of the labeled cRNAs were measured by NanoDrop. Each labeled cRNA was fragmented and hybridized to the microarray slide. The slides were incubated for 17 h at 65 °C in an Agilent Hybridization Oven. The hybridized arrays were washed, fixed, and scanned using the Agilent DNA Microarray Scanner. Agilent Feature Extraction Software v 11.0.0.1 was used to analyze acquired array images. Quantile normalization and subsequent data processing were performed using the GeneSpring GX v 12.0 software package (Agilent Technologies). After quantile normalization of the raw data, mRNAs that were flagged as present in all of the samples were chosen for further data analysis. Differentially expressed mRNAs with statistical significance were identified through Volcano Plot filtering. Microarray data are available through the Gene Omnibus Expression database, accession number GSE83659.

### qPCR

For analysis of mature miRNA expression, cDNA was synthesized using the miRCURY LNA Universal RT kit and added to qPCR reactions containing miRCURY LNA Primer Sets (Qiagen) specific for miR-22, or U6 and 2x Fast SYBR Green Master Mix (Thermo Fisher).

For analysis of primary miRNA, mRNA, or lncRNA expression, cDNA was synthesized using the RevertAid First Strand cDNA Synthesis Kit with random hexamer primers and used in qPCR reactions with TaqMan assays specific for human, rat and mouse *NEAT1*, pri-miR-22 and *GAPDH* (Thermo Fisher) and 2x Universal TaqMan Master Mix (Thermo Fisher). All qPCR reactions were run on a StepOnePlus Real-Time PCR System (Thermo Fisher) and Ct values were normalized first to those of their respective reference genes (*GAPDH* for mRNA and U6 RNA for miRNA expression), second to the mean of the control samples (ΔΔCt) and expressed using the formula 2^–ΔΔCt^.

### Profiling of miRNA:pri-miRNA expression

Custom TaqMan qPCR array plates including assays for the 96 miRNAs with highest expression in human cardiac tissue in Spengler et al.^20^ and their respective pri-miRNAs were acquired from Thermo Fisher. cDNA for miRNA or pri-miRNA analysis was prepared with TaqMan Advanced miRNA cDNA Synthesis Kit (Thermo Fisher) and High Capacity cDNA Synthesis Kit, respectively, using RNA from isolated from iPS-CM transfected with siNeat1 or siScr (*n* = 3 per group). miRNA and pri-miRNA expression levels were analyzed on a StepOne Plus qPCR instrument as described above. The signal from each assay was normalized to that of the global mean of all assays on the panel and expressed relative to the mean of the cells transfected with siScr. Differential expression was analyzed using multiple *t*-tests.

### FISH

A set of Quasar670-conjugated RNA FISH probes were designed for *NEAT1* using the Stellaris Probe Designer v. 4.2 and acquired from LGC Biosearch (Petaluma, CA, USA). RNA FISH was performed according to the manufacturer’s protocol. iPS-CM were seeded and cultured on glass coverslips in a 12-well plate. Five days after seeding, cells were fixed, permeabilized, and incubated with hybridization buffer containing 125 nM FISH probes overnight. The following day, cells were counterstained with 150 nM 4′,6-diamidino-2-phenylindole (DAPI) for 30 min, washed and mounted on microscopy slides with Diamond antifade mounting medium (Thermo Fisher) and visualized with a Nikon LU4A Ti Microscope (Nikon Instruments, Tokyo, Japan). The number of distinct *NEAT1* foci/cell was enumerated in >20 individual images from at least two different slides per condition, blinded to experimental groups.

### Flow cytometry

The proportion of apoptotic and necrotic cells was assessed with Annexin V and propidium iodide staining and flow cytometry. After treatment, cells were detached using Trypsin/EDTA, washed with PBS, and resuspended in Annexin V binding buffer (BD Biosciences, Franklin Lakes, NJ, USA). FITC-labeled Annexin V and propidium iodide (#556547, BD Biosciences) was added to cells followed by a 15 min incubation. The number of AnnV^+^/PI^−^ and AnnV^+^/PI^+^ events/μl were quantified using an Apogee Flow MICRO-PLUS Flow cytometer (Apogee Flow, Hemel Hempstead, UK).

### Caspase 3/7 activity assay

Cells were seeded in white 96-well plates at 20,000 cells/well and treated as described above. After treatment, cells were analyzed using the Caspase-Glo 3/7 assay (Promega, Madison, WI, USA) according to the manufacturer’s instructions with a CLARIOstar plate reader (BMG Labtech, Ortenberg, Germany).

### Lactate dehydrogenase release

Cells were seeded in 96-well plates at 20,000 cells/well and treated as described above. In all, 10 μl of cell medium was aspirated at indicated time points, diluted 1:10 with lactate dehydrogenase (LDH) Storage Buffer (200 mM Tris-HCl, pH 7.3, 10% Glycerol, 1% BSA), and stored at −20 °C until analysis. After thawing, samples were analyzed using the LDH-Glo Cytotoxicity Assay (Promega) according to the manufacturer’s instructions.

### Cleaved caspase 3 western blot

Proteins were extracted from iPS-CM by homogenization in RIPA buffer (Sigma Aldrich) containing protease inhibitor cocktail (Roche). For each sample, 10 µg of proteins were separated in reducing conditions by 4–12% SDS-PAGE and transferred to PVDF membrane (ThermoFisher Scientific). The membrane was blocked for 2 h at room temperature with 5% powdered skim milk in Tris-buffered saline pH 7.4 with 0.1% Tween (T-TBS). The membrane was then incubated with Apoptosis Western Blot Cocktail (#ab136812, Abcam, 1:250), or HSP90 (#610418, BD Biosciences, 1:3000). Antibodies were diluted in T-TBS with 3% bovine serum albumin (BSA, Sigma) and incubated overnight at 4 °C. Blots were washed five times with T-TBS and incubated with HRP-conjugated secondary antibodies anti-mouse (#12/2013, Cell Signaling Technology, Danvers, MA, USA, 1:10000) and anti-rabbit (#07/2014, Cell Signaling Technology, 1:10000) in T-TBS with 5% powered skim milk for 1 h at room temperature. Blots were washed five times with T-TBS. Bound antibodies were detected through enhanced chemiluminescence (Clarity Max, Bio-Rad, Hercules, CA, USA). Bands were analyzed by densitometry, using Fiji-ImageJ software.

### Primary miR-22 processing assay

A pri-miR-22 processing reporter was constructed according to the principles of a previous publication^21^. The mouse pri-miR-22 sequence (which displays complete sequence identity with human pri-miR-22) was cloned into the 3′UTR of the *Renilla* luciferase reporter psiCHECK-2 (Promega) by ligating the primers: priMir22CSU (5′-TCG AGG CCCT CAC CTG GCT GAG CCG CAG TAG TTC TTC AGT GGC AAG CTT TAT GTC CTG ACC CAG CTA AAG CTG CCA GTT GAA GAA CTG TTG CCC TCT GCC CCT GGC TTC GGC-3′) and primir22CSL (5′-GGC CGC CGA AGC CAG GGG CAG AGG GCA ACA GTT CTT CAA CTG GCA GCT TTA GCT GGG TCA GGA CAT AAA GCT TGC CAC TGA AGA ACT ACT GCG GCT CAG CCA GGT GAG GGC C-3′) into the Not1 and Xho1 sites of pSICheck2 (Promega). The firefly luciferase reporter signal was used for normalization and adjustment for transfection efficiency and differences in cell number. Luciferase activity was measured using the Dual-Luciferase Reporter Assay System (Promega) according to the manufacturer’s instructions and a CLARIOstar plate reader.

### RNA pull down

The T7 promoter was inserted into the NheI and BgIII sites of the pCMV-Neat1 expression vector containing the Neat1_1 isoform^22^. The miR-22 binding motif in *NEAT1* was deleted by inverse PCR, essentially as described by Hansson et al.^23^, using the deletion primers delmir22F (5′-AAG GAG ACT AGA TTT TGG GCC AAA GCG CCT TTA ACA ATC C-3′) and delmir22R 5′-TTA AAG GCG CTT TGG CCC AAA ATC TAG TCT CCT TTA TAC-3′) to produce the pCMV-Neat1Δm22 vector. Biotinylated probes were produced by in vitro transcription using linearized pCMV-Neat1 and pCMV-Neat1Δm22 vectors and the MEGAscript T7 Kit (Thermo Fisher) according to the manufacturer’s instructions. Successful biotinylation of in vitro transcribed RNA was confirmed with Bioanalyzer (Agilent, Santa Clara, CA, USA). In all, 3 μg of biotinylated Neat1 probe was denatured at 90 °C for 2 min, then placed on ice for 2 min. The RNA was then incubated for 20 min in RNA structure buffer (10 mM Tris, 0.1 M KCl, and 10 mM MgCl_2_). Nuclear extracts were prepared by resuspension of HL-1 cells in nuclear isolation buffer (1.28 M sucrose, 20 mM MgCl_2_, and 4% Triton-X 100) for 20 min before centrifugation at 2500 × *g* for 15 min and resuspension in RIP Buffer (150 mM KCl, 25 mM Tris, 0.5 mM DTT, 0.5% NP-40, 1 mM PMSF, and protease inhibitors). Nuclei were then sheared in a Dounce homogenizer and the nuclear extract was cleared by centrifugation at 13,000 × *g* for 10 min. In all, 5% of the volume was removed as input control and the remainder was incubated together with the Neat1 probe for 1 h at room temperature. Streptavidin-conjugated Dynabeads, pre-coated for 2 h with 1 mg/ml BSA and 1 mg/ml Yeast tRNA, were then added to the reaction and incubated for 1 h at room temperature. Dynabeads were then isolated using a magnetic tube rack and washed twice in nuclear isolation buffer, three times in low salt buffer (0.1% SDS, 1% Triton-X 100, 2 mM EDTA, 20 mM Tris-HCl, and 150 mM NaCl) and once in high salt buffer (0.1% SDS, 1% Triton-X 100, 2 mM EDTA, 20 mM Tris-HCl, and 500 mM NaCl). QIAzol was then added to the beads and RNA was isolated using the miRNeasy mini kit. Dynabeads mixed with nuclear extract (no probe) was included as a negative control. An equal volume of RNA preparation and 5% Input control was used for cDNA-synthesis with the RevertAid H- First Strand Synthesis Kit and random hexamer primers. The amount of precipitated pri-miR-22 was analyzed with qRT-PCR as described above and expressed relative to the Input control.

### Statistical methods

Data are presented as mean and standard deviation. Statistical significance between experimental groups was assessed with Student’s *t* tests or one-way ANOVA with Sidak’s test for multiple comparisons as applicable. For microarray data, adjustment of *p*-values for multiple comparisons was performed according to the principle of false discovery rate. The threshold for statistical significance were *p* < 0.05 or *q* < 0.05. Statistical tests are specified for each experiment in the figure legends. All data were deemed to be normally distributed and the variance of data was comparable between groups. Sample size in vivo and in vitro was chosen based on extensive experience with the IPC model, cardiac gene expression, and cardiomyocyte biology. No animals were excluded from the analysis. No randomization of animals was performed. Investigators were not blinded to the group allocation in vivo or in vitro during or after the experiment. All statistical analyses were performed in GraphPad Prism version 8.1.1 (GraphPad Software, San Diego, CA, USA).

## Results

### IPC results in rapid and distinct downregulation of Neat1

**Fig. 1:**
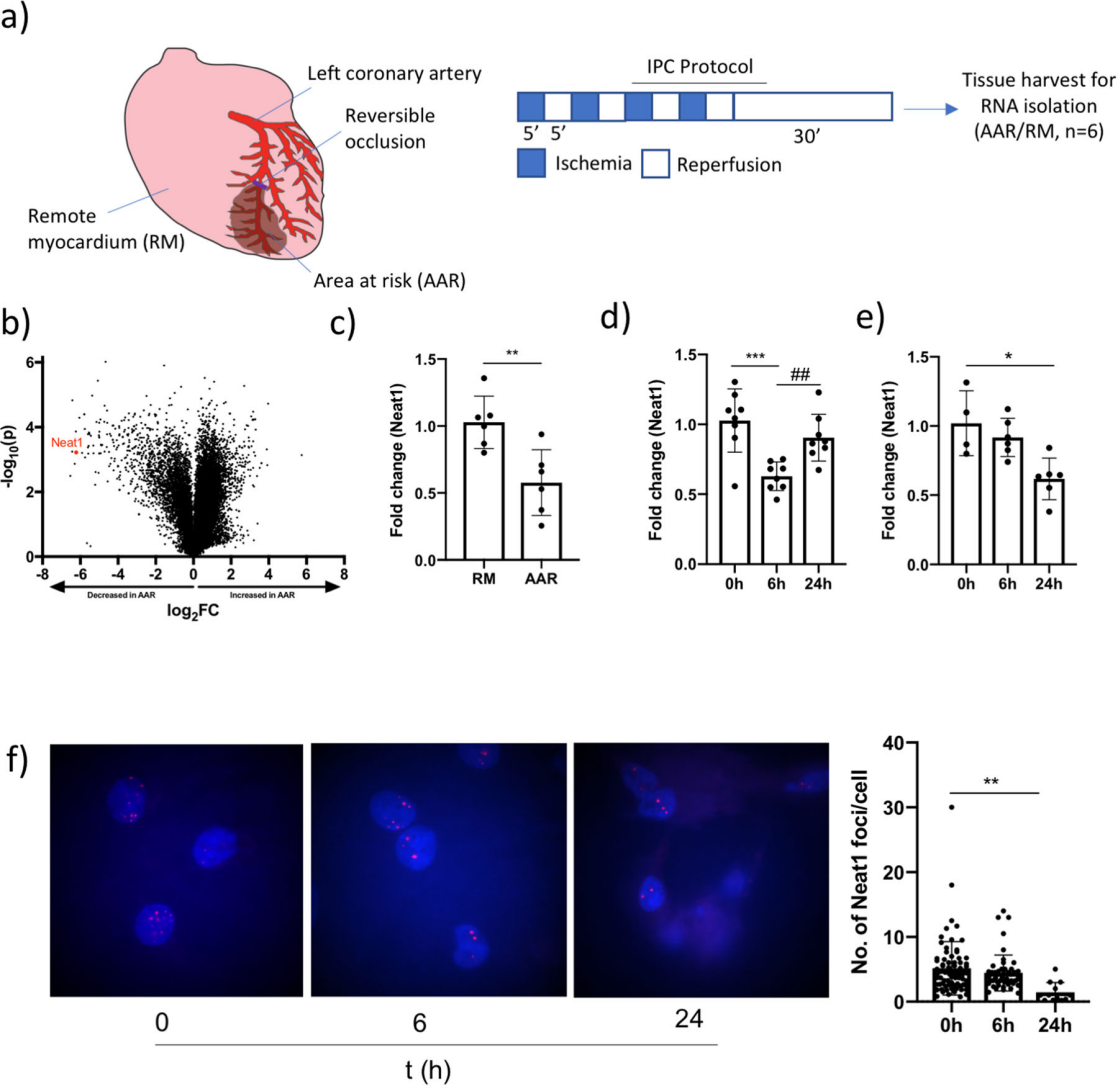
Profiling of cardiac lncRNAs in an ischemic preconditioning model. **a** Principles of the murine ischemic preconditioning (IPC) model. Brief repeated periods of ischemia/reperfusion are achieved by reversible occlusion of the left coronary artery and RNA is isolated from the area at risk (AAR) and remote myocardium (RM) following IPC and 30 min of reperfusion. **b** Volcano plot comparing the expression of 31,423 lncRNA in RM and AAR from IPC mice (*n* = 2 in each group). *Neat1* is indicated in red. **c** Expression of *Neat1* in the AAR and RM of IPC mice (*n* = 6 per group) analyzed by qRT-PCR, normalized to *Gapdh* and expressed relative to the mean of the RM samples. **p < 0.01. Expression of *NEAT1* in **d** human iPS-derived cardiomyocytes (IPS-CM) and **e** rat neonatal cardiomyocytes during hypoxia (1% pO_2_) analyzed by qRT-PCR, normalized to *GAPDH* and expressed relative to the mean of the baseline (0 h) samples. Results are based on two separate experiments with 2–4 biological replicates in each group. *p < 0.05 comparing the 0 h and 24 h timepoints in rat neonatal cardiomyocytes. ***p < 0.001 comparing the 0 h and 6 h timepoints in iPS-CM. ##<0.01 comparing the 6 h and 24 h timepoints in iPS-CM. **f** RNA FISH for *NEAT1* in iPS-CM during hypoxia (1% pO_2_). Left, representative fluorescence microscope images of cells at each time point. *NEAT1* foci are shown in red and nuclei were counterstained with DAPI (blue). Right, quantitation of *NEAT1* foci per cell at each time point. >12 images were analyzed per group. **p < 0.01 comparing 0 h and 24 h. All graphs show mean and standard deviation.

To identify non-coding transcripts involved in IPC-induced cardioprotection, we performed an exploratory profiling of 31,423 lncRNAs in the area at risk (AAR) and remote myocardium (RM) from mice 30 min after IPC (*n* = 2) with microarray (Fig. 1a). In all, 2702 lncRNAs met the criteria for differentially expressed transcripts (FDR-adjusted *p*-value < 0.05 and a fold change between AAR and RM > 2), of which 1559 were increased and 1143 were decreased in the AAR (Fig. 1b). We surveyed the lncRNAs with the most pronounced differences between AAR and RM (Table 1) and identified *Neat1* as one of the most interesting candidates for validation and mechanistic follow-up. *Neat1*, a nuclear RNA and an essential component of paraspeckles^24^ was one of the most distinctly downregulated lncRNAs in the AAR (decreased ~80-fold compared to RM). *Neat1* has recently been reported to suppress cardiomyocyte apoptosis^25^ and exacerbate myocardial injury in response to ischemia reperfusion^26^. Thus, we hypothesized that the rapid and forceful downregulation of *Neat1* following IPC could be part of a cardioprotective mechanism. To confirm the effect of IPC on Neat1 expression, qPCR was performed on AAR and RM biopsies from a separate group of mice and a ~50% decrease was observed (*p* < 0.01, *n* = 5–6, Fig. 1c).

**Table 1:** Top differentially regulated lncRNAs.

Transcript ID	Gene Symbol	Source	Transcript length (nt)	Type	Associated gene symbol	Mean AAR	Mean RM	Fold change (AAR vs RM)	Adjusted *p*-value
*Increased in AAR*									
ENSMUST00000127786	Xist	Ensembl	17,041	Intergenic	–	10053.44	187.61	53.59	0,019
AK030243		fantom3	2295	Intergenic	–	1001.42	63.87	15.68	0,004
AK035076		fantom3	2397	Intergenic	–	318.67	20.97	15.20	0,035
ENSMUST00000153883	Xist	Ensembl	626	Intergenic	–	746.60	50.04	14.92	0,020
ENSMUST00000160241	Gm15810	Ensembl	681	Antisense overlap	Phactr1	526.82	43.02	12.25	0,019
uc009bpe.1	TCR-beta chain	UCSC_kg	740	Intergenic	–	708.86	61.62	11.50	0,036
ENSMUST00000133015	Gm12542	Ensembl	647	Antisense overlap	Snx30	351.72	33.75	10.42	0,048
AK087032		fantom3	2311	Intergenic	–	3904.02	392.42	9.95	0,005
uc009iqv.1	Art1	UCSC_kg	2341	Antisense overlap	Chrna10	1826.98	187.41	9.75	0,004
AK021184		NRED	703	Antisense overlap	Adam10	2086.07	214.43	9.73	0,021
*Decreased in AAR*									
ENSMUST00000120647	Gm11839	Ensembl	1953	Intergenic	–	20.74	1917.31	0.011	0,035
AK050743		fantom3	2885	Intergenic	–	63.26	5466.28	0.012	0,018
ENSMUST00000121449	Gm15502	Ensembl	208	Sense overlap	Cyp3a13	445.75	38278.28	0.012	0,003
ENSMUST00000145087	Gm6706	Ensembl	318	Intergenic	–	45.86	3602.22	0.013	0,008
ENSMUST00000120853	Gm11400	Ensembl	1214	Sense overlap	Sfi1	51.71	3894.68	0.013	0,004
NR_003513	Neat1	RefSeq_NR	3177	Intergenic	–	58.38	4353.39	0.013	0,015
ENSMUST00000162504	Gm8459	Ensembl	760	Intergenic	–	87.79	5808.41	0.015	0,000
ENSMUST00000120973	Gm5379	Ensembl	826	Intergenic	–	57.67	3463.26	0.017	0,019
uc007zya.1	Son	UCSC_kg	10879	Sense overlap	Son	26.29	1453.14	0.018	0,001
ENSMUST00000122299	Gm15466	Ensembl	333	Sense overlap	Mrps28	253.89	13834.26	0.018	0,005

Prolonged ischemia was reported to result in increased *Neat1* expression in cardiac tissue and cardiomyocytes^25,26^. We wanted to investigate whether short-term hypoxia instead constitutes a conserved trigger for *Neat1* downregulation in cardiomyocytes by culturing primary rat neonatal cardiac myocytes and human iPS-derived cardiomyocytes at 1% pO_2_ and analyzing *Neat1* expression over time. We observed a significant decrease in *Neat1* expression in both cell types but with slightly different dynamic patterns. In iPS-CM, *NEAT1* expression was significantly decreased 6 h after hypoxia (*p* < 0.01 compared to 0 h), but with expression levels returning towards baseline at 24 h (*p* < 0.01 compared to 6 h, Fig. 1d). In rat cardiomyocytes, there was a gradual decrease in *Neat1* expression that resulted in a significantly lower expression level at 24 h (*p* < 0.05, Fig. 1d). In addition, we assessed the number of nuclear *NEAT1* foci (indicative of paraspeckles) in iPS-CM with FISH and observed a gradual decrease during hypoxia (Fig. 1f).

### Neat1 knock down protects cardiomyocytes from hypoxia

**Fig. 2:**
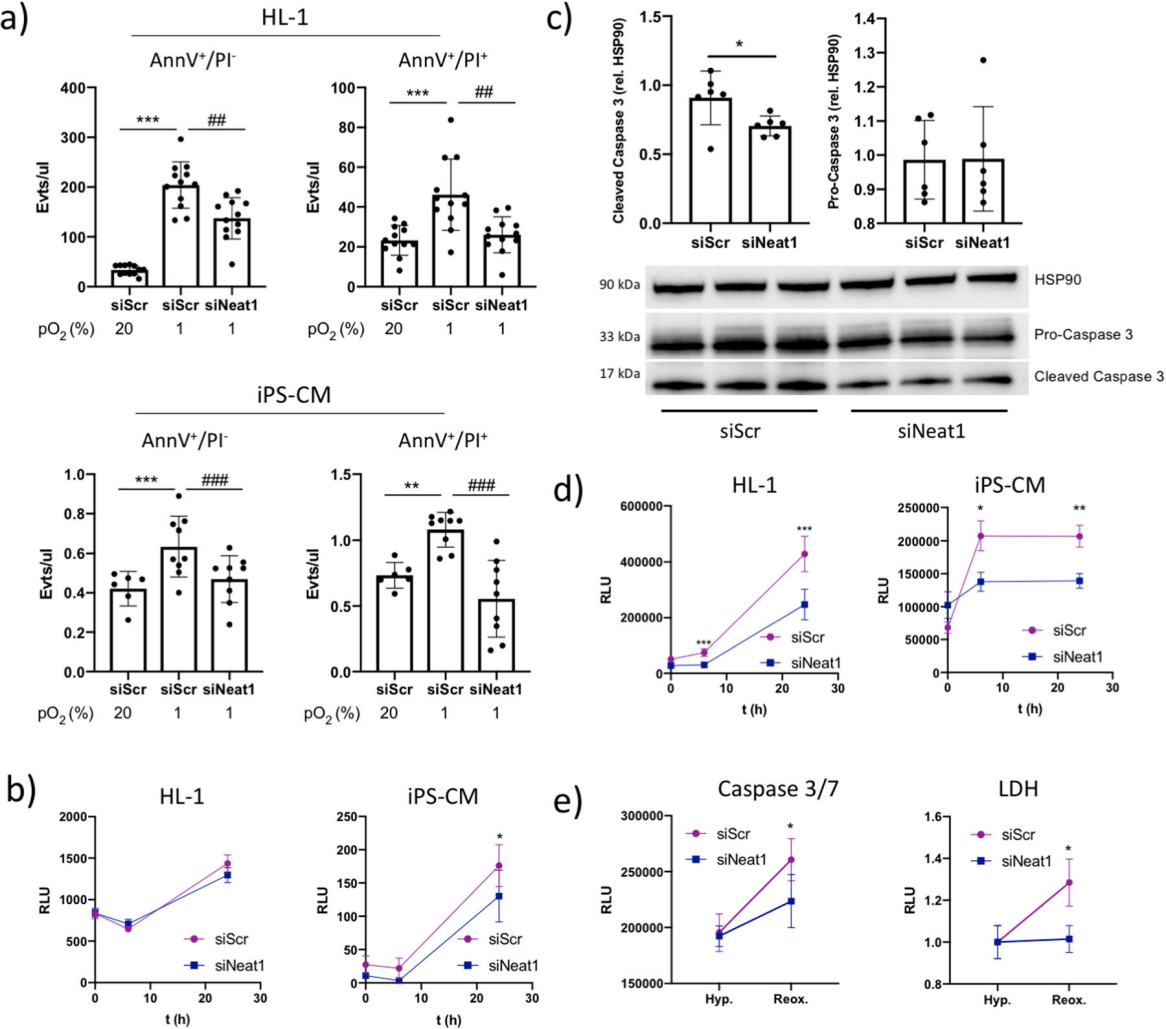
Neat1 knock down confers protection to hypoxia in murine and human cardiomyocytes. **a** Annexin V/propidium iodide staining of HL-1 and iPS-CM after transfection with scrambled control siRNA (siScr) or siRNA to Neat1 (siNeat1) and grown under normoxic or hypoxic (1% pO_2_) conditions for 24 and 6 h, respectively. The number of AnnV^+^/PI^−^ and AnnV^+^/PI^+^ per μl was quantified with flow cytometry. Results are based on two separate experiments with six replicates in each group. **p < 0.01, ***p < 0.001 comparing cells transfected with siScr under normoxic and hypoxic conditions. ##*p* < 0.01, ###*p* < 0.001 comparing cells transfected with siScr and siNeat1 under hypoxic conditions. **b** Quantification of Caspase 3/7 activity in HL-1 and iPS-CM transfected with siScr or siNeat1 and grown in hypoxic conditions for 24 h. Results are based on two separate experiments with three replicates in each group. *p < 0.05 comparing cells transfected with siScr and siNeat1. **c** Quantification of pro-caspase 3 and cleaved caspase 3 in iPS-CM transfected with siScr and siNeat1. Protein was isolated from transfected cells cultured in hypoxia for 6 h and used for western blot. The amount of pro-caspase 3 and cleaved caspase 3 was quantified relative to HSP90, *p < 0.05. **d** Quantification of LDH in the cell medium of HL-1 and iPS-CM transfected with siScr or siNeat1 and grown in hypoxia for 24 h. *p < 0.05, **p < 0.01, ***p < 0.001 comparing cells transfected with siScr and siNeat1 at each time point. **e** Caspase 3/7 activity and LDH release in iPS-CM transfected with siNeat1 or siScr and subjected to 24 h of hypoxia or 24 h of hypoxia followed by 6 h of reoxygenation. *p < 0.05 comparing hypoxia with hypoxia/reoxygenation. RLU relative luminescence units. All graphs show mean and standard deviation.

To assess whether cardiac *Neat1* downregulation confers protection from hypoxia, we transfected the murine cardiac cell line HL-1 and iPS-CM with siRNA targeting *Neat1* (siNeat1) or scrambled control siRNA (siScr) before subjecting the cells to hypoxia (1% pO_2_). Successful knock down of *Neat1* expression was confirmed with qPCR (Supplementary Fig. 1). Cells were stained with Annexin V (AnnV) and Propidium Iodide (PI) and the proportion of apoptotic (AnnV^+^/PI^−^) and necrotic (AnnV^+^/PI^+^) cells was analyzed with flow cytometry (Fig. 2a). As expected, hypoxia caused a significant increase in AnnV^+^/PI^−^ and AnnV^+^/PI^+^ cells, but this effect was effectively reversed in cells transfected with siNeat1. To corroborate these findings, Caspase 3/7 activity was monitored during 24 h in HL-1 and iPS-CM transfected with siNeat1 or siScr and cultured under hypoxic (1% pO_2_) conditions (Fig. 2b). Hypoxia-induced elevation of Caspase activity was significantly inhibited by *Neat1* knock down in iPS-CM (*p* < 0.001), but to a lesser extent in HL-1 (*p* > 0.05). To confirm the effect of *Neat1* knock down on hypoxia-induced Caspase 3 activity, levels of pro-Caspase 3 and cleaved Caspase 3 were quantified in iPS-CM using western blot. Cells transfected with siNeat1 had significantly less cleaved Caspase 3 in response to hypoxia than cells transfected with siScr (*p* < 0.05). Finally, LDH release was quantified in the cell medium of HL-1 and iPS-CM transfected with siNeat or siScr and subjected to hypoxia for 24 h. A significant increase in LDH was observed in IPS-CM and HL-1 after 6 and 24 h, respectively, but levels were significantly lower in the medium of cells transfected with siNeat1. Taken together, we conclude that decreased *Neat1* expression renders both murine and human cardiomyocytes less sensitive to hypoxia. To assess whether *Neat1* knock down would also have a beneficial effect on ischemia/reperfusion induced cardiomyocyte apoptosis and necrosis, we transfected iPS-CM with siNeat1/siScr and subjected them to prolonged (24 h) hypoxia followed by a period (6 h) of reoxygenation. There was a significant increase in both Caspase 3/7 activity and LDH release upon reoxygenation in control cells, but this effect was attenuated or absent in cells transfected with siNeat1.

### Neat1 regulates the processing of pri-miR-22

**Fig. 3:**
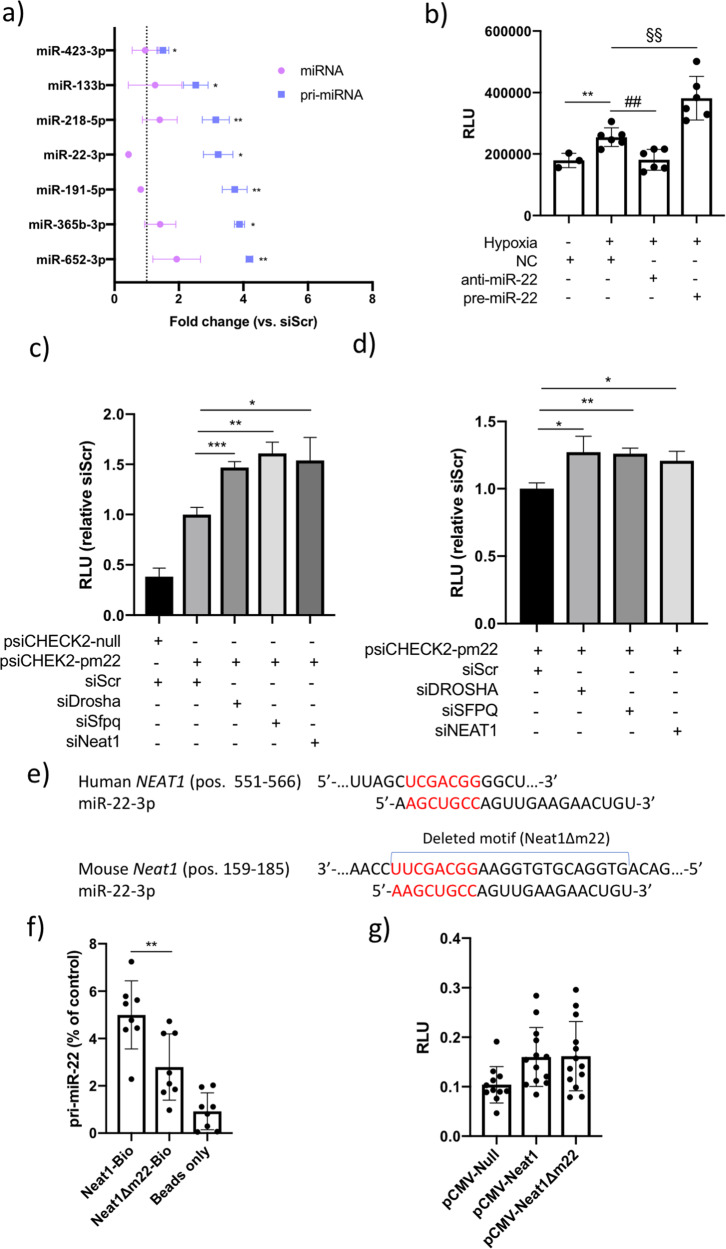
Neat1 regulates processing of the pro-apoptotic microRNA miR-22 in cardiomyocyte nuclei. **a** Expression of miRNAs and their respective pri-miRNA in iPS-CM transfected with siNeat1 analyzed with qRT-PCR. Results are expressed relative to cells transfected with negative control siRNA (siScr). **/*p < 0.01/p < 0.05 comparing pri-miR expression in cells transfected with siNeat1 compared to cells transfected with siScr. **b** Quantification of Caspase 3/7 activity in iPS-CM transfected with negative control miRNA (NC), anti- or pre-miR-22 and grown in normoxic or hypoxic conditions. **p < 0.01 comparing cells transfected with NC in normoxic and hypoxic conditions. ##*p* < 0.01 comparing cells transfected with NC and anti-miR-22. §§*p* < 0.01 comparing cells transfected with NC and pre-miR-22. **c**, **d** Pri-miR-22 processing reporter assay in HL-1 cells and iPS-CM, respectively. The pri-miR-22 sequence was cloned into the 3′UTR of a *Renilla* luciferase reporter vector (psiCHECK2-pm22) so that the luciferase signal is inversely correlated with pri-miR-22 processing activity. The vector was co-transfected with negative control siRNA (siScr) or siRNA to *Drosha*, *Sfpq*, or *Neat1* into HL-1 or iPS-CM. ***p < 0.001, **p < 0.01, *p < 0.05. Results are normalized to the mean of the signal in cells transfected with siScr. Data are derived from two separate experiments with 3–6 replicates in each group. **e** Representation of miR-22 binding sites in human and mouse *Neat1*. Complementary nucleotides within the miR-22 seed sequence are shown in red. The deleted motif in the Neat1Δm22 vector is indicated with a capped blue line. **f** Levels of co-precipitated pri-miR-22 using an intact Neat1 probe, the Neat1Δm22 probe or streptavidin beads alone assessed with qRT-PCR. Results are expressed in relation to total input RNA. Data are derived from three separate experiments with 2–3 replicates in each group. **g** Pri-miR-22 processing reporter assay in HL-1 cells co-transfected with empty expression vector (pCMV-Null), vector containing intact *Neat1* (pCMV-Neat1), or *Neat1* without the miR-22 binding motif (pCMV-*Neat1*Δm22). Results are from two separate experiments with 5–6 replicates in each group. All graphs show mean and standard deviation.

Next, we wanted to investigate a possible mechanism by which *Neat1* downregulation in cardiomyocytes leads to increased tolerance to hypoxia. The decrease in nuclear *NEAT1* foci in response to hypoxia pointed towards a mechanism involving the role of the lncRNA as a structural and functional component of nuclear paraspeckles. Recently, Jiang et al.^21^ reported that *NEAT1* facilitates efficient processing of primary microRNA (pri-miRNA) transcripts through interaction with the paraspeckle components NONO and SFPQ and recruitment of the Drosha-DGCR8 Microprocessor complex. We wanted to assess to what extent pri-miRNA processing was regulated by *NEAT1* in human cardiomyocytes. To this end, we performed a comprehensive analysis of miRNAs with previously reported^20^ high cardiac expression and their respective pri-miRNAs in iPS-CM after *NEAT1* knock down. We assumed that for miRNAs that are dependent on *NEAT1* for efficient processing, knock down would lead to significantly increased levels of pri-miRNA while the mature miRNA would be decreased or unaffected. Of the 96 miRNAs in the analysis, seven such pri-miRNA/miRNA-pairs were identified: miR-423-3p, miR-133b, miR-218-5p, miR-22-3p, miR-191-5p, miR-365-3p, and miR-652-3p (Fig. 3a). These results points towards a role for *NEAT1* in the processing of specific pri-miRNAs in cardiomyocytes. We were particularly interested in pri-miR-22/miR-22-3p given that it is among the most abundant miRNAs in cardiac tissue^20^ as well as in iPS-CM (Supplementary Fig. 2), and has previously been reported to contribute to myocardial ischemia/reperfusion injury^27,28^. We thus hypothesized that the interaction between Neat1 and pri-miR-22 could be part of a cardioprotective mechanism.

First, we sought to confirm a pro-apoptotic role for miR-22 in iPS-CM. Overexpression of miR-22 was achieved by transfection of iPS-CM with a synthetic pre-miR-22 molecule and apoptosis was analyzed after 24 h using a Caspase 3/7 assay. A 7-fold increase in miR-22 expression was observed in cells transfected with pre-miR-22 compared to control (Supplementary Fig. 3a). As expected, increased miR-22 expression led to a substantial increase in Caspase 3/7 activity (*p* < 0.01, Supplementary Fig. 3b). We next tested the role of miR-22 in the response of cardiomyocytes to hypoxia by modulating miR-22 expression in iPS-CM via synthetic pre- and anti-miR-22, respectively, and assessed the level of apoptosis via a Caspase 3/7 activity assay at 24 h. Hypoxia-induced apoptosis was reversed by inhibition of miR-22 expression and exacerbated by overexpression of miR-22 in iPS-CM (Fig. 3b).

We next wanted to confirm that *Neat1* influences the processing of pri-miR-22 in human and murine cardiomyocytes using a reporter construct where the pri-miR-22 sequence was inserted into the 3′UTR of the Renilla Luciferase reporter (denoted psiCHECK-pm22). A decrease in pri-miR-22 processing would therefore lead to increased luciferase activity. We co-transfected HL-1 cells with the psiCHECK-pm22 vector and siRNA towards *Neat1* or *Sfpq* into HL-1 cells and measured luciferase activity. As a positive control, one group of cells were co-transfected with the reporter construct and siRNA for *Drosha*: the dsRNA nuclease that cleaves pri-miRNA and initiates miRNA processing. Expectedly, siDrosha caused a significant increase in reporter activity compared to siScr (*p* < 0.001). Knock down of either Neat1 or *Sfpq* also resulted in a similar increase in luciferase activity (*p* < 0.05 and *p* < 0.01, respectively), indicating that *Neat1* and paraspeckles are involved in the processing of pri-miR-22 in cardiomyocytes. To test whether this mechanism was conserved in human cells, we repeated the experiment in iPS-CM (Fig. 3d) and observed similar results.

In the report by Jiang et al., the authors did not investigate whether base pairing between *Neat1* and pri-miRNAs was required for efficient processing. We identified potential miR-22 binding sites in both mouse and human *NEAT1* (Fig. 3e). To confirm the association between *Neat1* and pri-miR-22 in cardiomyocytes, we repeated the RNA pull down experiment with a probe where the miR-22 target site had been deleted. We observed a distinct decrease in the amount of precipitated pri-miR-22 with the mutated probe (Fig. 3f), which indicates that *Neat1* does directly bind to pri-miR-22 in the nuclei of mouse cardiomyocytes. To analyze whether this binding is required for pri-miR-22 processing, we co-transfected HL-1 cells with the psiCHECK-pm22 and an expression vector containing either the intact *Neat1* (pCMV-Neat1) or *Neat1* without the miR-22 target site (pCMV-Neat1Δm22). Luciferase activity was comparable between cells transfected with pCMV-Neat1 and pCMV-Neat1Δm22 (Fig. 3g), meaning that although pri-miR-22 binds to *Neat1*, this association is not required for efficient processing.

**Fig. 4:**
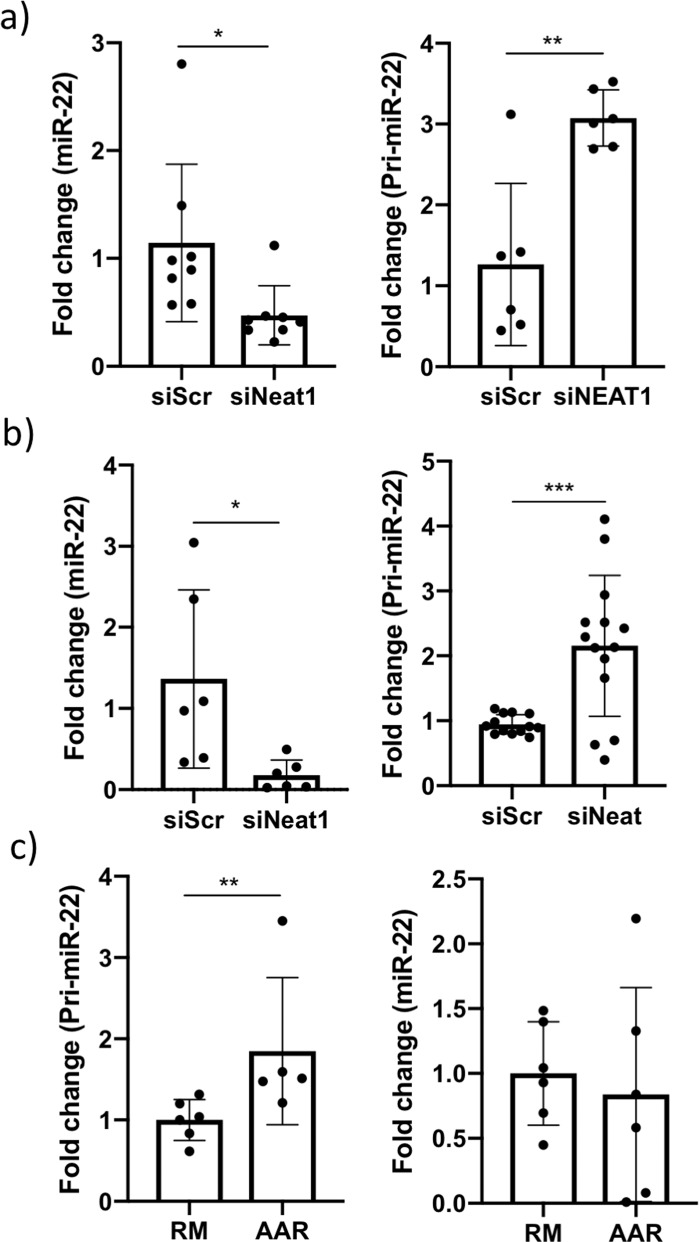
Knock down of Neat1 results in an accumulation of unprocessed primary miR-22. **a** qRT-PCR analysis of miR-22 and pri-miR-22 in **a** HL-1 cells and **b** iPS-CM transfected with siRNA to *Neat1* and **c** in the RM and AAR of IPC mice. miR-22 and pri-miR-22 levels are expressed relative U6 RNA and *Gapdh*, respectively. Results are normalized to the mean of the cells transfected with negative control siRNA or to the RM samples in the case of the IPC mice. *p < 0.05, **p < 0.01, ***p < 0.001. All graphs show mean and standard deviation.

To confirm the link between *Neat1* and pri-miR-22 processing, we analyzed the levels of pri-miR-22 and mature miR-22 in HL-1 cells and iPS-CM upon *Neat1* knock down. As expected, we observed a marked accumulation of pri-miR-22 and a depletion of mature miR-22 in both human (Fig. 4a) and mouse cardiomyocytes (Fig. 4b) after *Neat1* knock down. Interestingly, pri-miR-22 was also significantly elevated in the AAR of IPC mice while the levels of mature miR-22 was unaffected (Fig. 4c).

### Hypoxia transiently inhibits pri-miR-22 processing

**Fig. 5:**
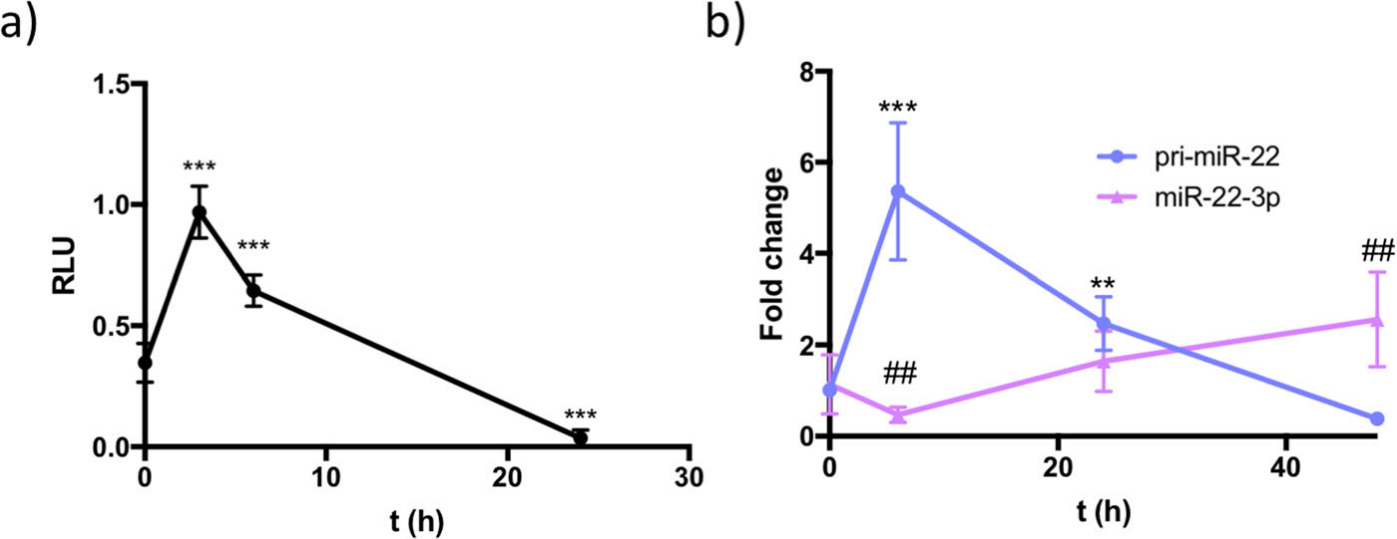
Hypoxia results in transient disruption of cardiomyocyte pri-miR-22 processing. **a** Pri-miR-22 processing reporter assay signal in iPS-CM grown in hypoxic conditions (1% pO_2_). ***p < 0.001 comparing the signal to baseline. **b** qRT-PCR analysis of pri-miR-22 and miR-22-3p expression in iPS-CM grown in hypoxic conditions. miR-22-3p and pri-miR-22 levels are expressed relative U6 RNA and *GAPDH*, respectively. Results are normalized to the mean of the baseline (0 h) samples. ***p < 0.001, **p < 0.01 comparing pri-miR-22 expression at time point 6 h and 24 h with time point 0 h. ##*p* < 0.01 comparing miR-22-3p expression at time point 6 h and 48 h with time point 0 h. All graphs show mean and standard deviation.

Given the role for *Neat1* in facilitating pri-miR-22 processing in cardiac nuclei and the decreased expression levels of *Neat1* during hypoxia, we hypothesized that hypoxia would lead to disturbed pri-miR-22 processing. iPS-CM were transfected with the psiCHECK-pm22 reporter construct and subjected to 24 h of hypoxia (1% pO_2_). The reporter signal peaked at 3 h after induction of hypoxia, stayed elevated at 6 h and decreased below the baseline value at 24 h (Fig. 5a). This suggests that hypoxia leads to transient inhibition of pri-miR-22 processing. This finding was corroborated by pri-miR-22 and mature miR-22 expression levels in iPS-CM at 6, 12, 24, and 48 h after hypoxia. There was a pronounced accumulation of pri-miR-22 at 6 h while the levels fell below the baseline at 48 h (Fig. 5b). In contrast, the levels of miR-22-5p expression (Fig. 5b) was downregulated at 6 h and then gradually increased at the later time points, showing a significant increase at 48 h compared to baseline. Taken together, these results support a mechanism whereby hypoxia leads to a rapid, transient decrease in pri-miR-22 processing in cardiomyocytes.

### The anti-apoptotic effect of Neat1 knock down is reversed by miR-22

**Fig. 6:**
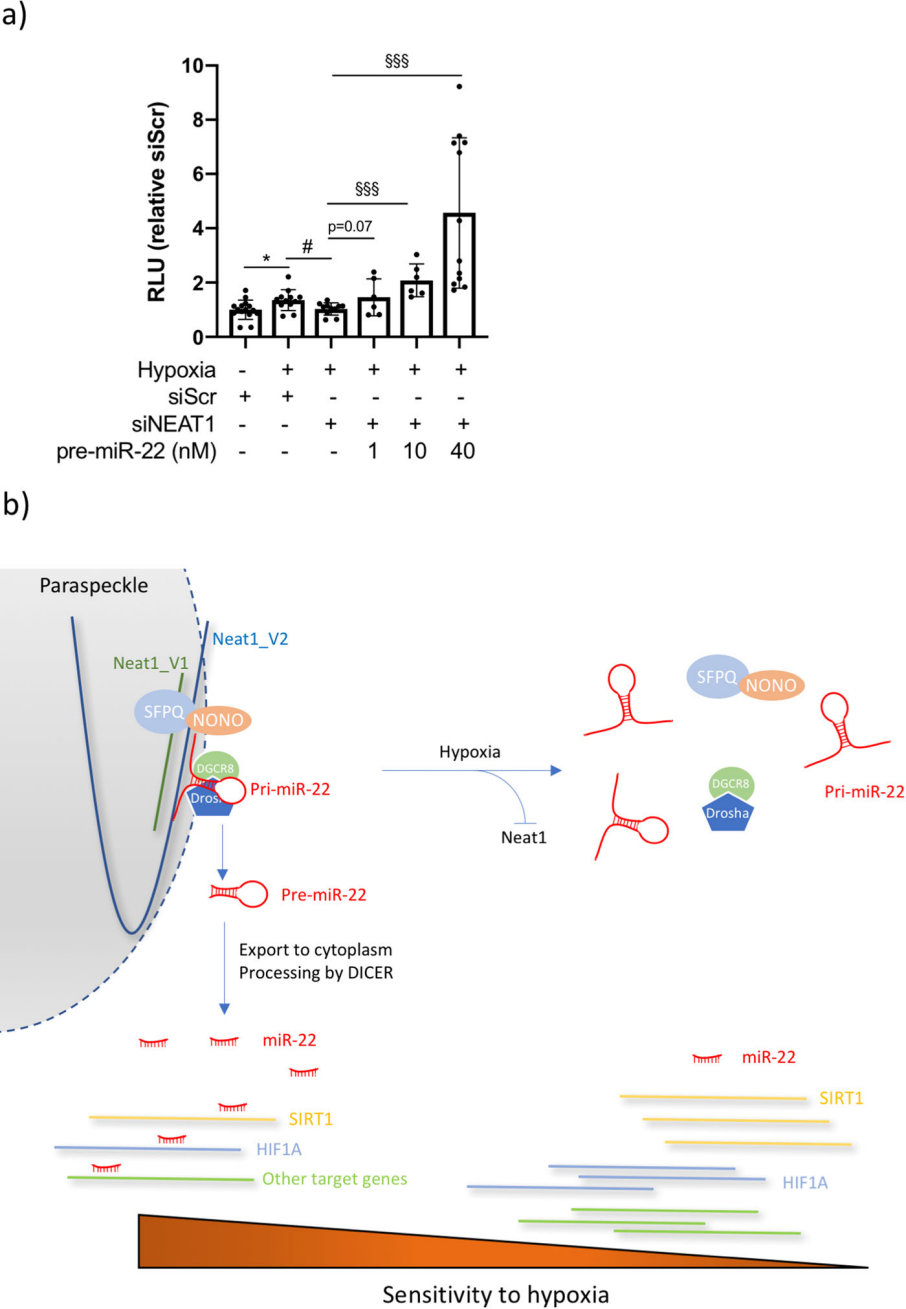
The resistance to hypoxia conferred by Neat1 knock down can be reversed by increasing the levels of mature miR-22. **a** Quantification of Caspase 3/7 activity in iPS-CM transfected with siScr or siNEAT1 and increasing doses of pre-miR-22 and grown in hypoxic conditions. *p < 0.05 comparing cells transfected with siScr and grown in normoxic and hypoxic conditions. #*p* < 0.05 comparing cells transfected with siScr and siNEAT1 in hypoxia. §§§*p* < 0.001 comparing cells transfected with siNEAT1 with and without 1, 10, or 40 nM pre-miR-22, respectively. Results are based on three separate experiments with 3–6 replicates in each group. The mean and standard deviation for each group is indicated. **b** Proposed mechanism by which *Neat1* indirectly affects the sensitivity of cardiomyocytes to hypoxia. The two Neat1 isoforms (denoted Neat1_V1 and Neat1_V2) form the structural basis of paraspeckles together with NONO/SFPQ heterodimers. *Neat1* recruits the Microprocessor complex (formed by DGCR8 and DROSHA) according to the model described by Jiang et al. and is required for efficient processing of pri-miR-22 in cardiomyocytes. Decreased *Neat1* levels in response to hypoxia leads to disruption of paraspeckles and an accumulation of unprocessed pri-miR-22 in the nucleus. The subsequent decrease in biologically active miR-22 in the cytoplasm renders cardiomyocytes less sensitive to hypoxia, possibly through derepression of validated mRNA targets such as *SIRT1* and *HIF1A*.

As the level of miR-22 appears to tune the sensitivity of cardiomyocytes to hypoxia, and the availability of mature miR-22 in the cytoplasm is dependent on *Neat1*, we hypothesized that the protective effect of *Neat1* knock down could be reversed by addition of exogenous miR-22. To test this, we co-transfected iPS-CM with *Neat1* siRNA and increasing doses of synthetic pre-miR-22 and analyzed Caspase 3/7 activity at 24 h of hypoxia. As seen in Fig. 6a, the anti-apoptotic effect of *Neat1* knock down was indeed reversed in a dose-dependent manner by addition of exogenous pre-miR-22. In the light of these findings, we propose a model where Neat1 confers indirect protection from hypoxia via its role in regulating pri-miR-22 processing in cardiomyocyte nuclei (Fig. 6b).

## Discussion

Given the multi-faceted roles of lncRNAs in cardiomyocyte biology in general^29^ and in the response to hypoxia specifically^30–32^, we hypothesized that this class of non-coding transcripts would contribute to IPC-induced cardioprotection. We performed a comprehensive exploratory analysis of lncRNA expression (>30,000 transcripts) in cardiac tissue from mice subjected to IPC and quite surprisingly, we observed a considerable perturbation of the lncRNA transcriptome. >2000 transcripts were either significantly increased or decreased already at 30 min after the treatment. These results points to a profound effect of IPC on epigenetic and transcriptional mechanisms in cardiomyocytes, and provides a wealth of clues as to the molecular mechanisms underlying the second window of cardioprotection. Among the most dramatically perturbed lncRNAs in cardiac tissue in response to IPC was *Neat1*, a lncRNA with a well-established role as a structural component of nuclear paraspeckles. It is worth noting that there was a distinct discrepancy between the expression levels observed in the microarray analysis and that of the qPCR-based validation in a separate group of animals. While the array results showed an 80-fold reduction in Neat1 in the AAR compared to RM, qPCR analysis showed a more modest 2-fold reduction. Gene expression levels generally correspond well between arrays and qRT-PCR^33^, but it has been shown that the correlation is less pronounced for downregulated genes due to increased variability in low-intensity array spots^34^. In addition, effects of dye biases, non-specific and/or cross hybridizations of labeled targets to probes, distance between the location of PCR primers and array probes are all factors that can distort array results and cause discrepancies with results from qRT-PCR. These discrepancies, together with the fact the number of biological replicates was low, mean that the extent to which the transcriptome profile is affected by IPC must be associated with a degree of uncertainty.

The rapid and marked decrease in Neat1 expression as well as the number of paraspeckles in response to brief hypoxia was replicated in human iPS-derived cardiomyocytes in vitro, pointing toward a conserved mechanism. This is to our knowledge the first time *NEAT1* and paraspeckles have been studied in human cardiomyocytes. We observed a discrepancy between the number of *NEAT1* nuclear foci and the transcription levels of *NEAT1* in iPS-CM in response to hypoxia. Whereas *NEAT1* transcription rebounded after 24 h, *NEAT1* nuclear foci continued to decrease at this time point. The reason for this discrepancy might be a delay in the assembly and organization of paraspeckles after the initial decrease in *NEAT1* expression. It is also possible that hypoxia has additional effects on the transcription or translation of other paraspeckle components, resulting in a gradual decrease in *NEAT1* FISH foci but not affecting *NEAT1* transcription.

We present evidence for a protective feedback mechanism in human iPS-derived cardiomyocytes whereby decreased *NEAT1* levels leads to impaired pri-miR-22 processing and protection from hypoxia. Support for the fact that *NEAT1* acts as a scaffold for the Microprocessor complex and thereby facilitating the processing of primary miRNA in HeLa cells was recently published^21^. In the present study, we provide evidence that this also occurs in murine and human cardiomyocytes. Although *NEAT1* knock down did not result in the general depletion of mature miRNAs seen in HeLa cells, we did observe an accumulation of a specific subset of pri-miRNA in cardiomyocytes. It is possible that the extent to which *NEAT1* influences pri-miRNA processing is cell type dependent, and a more detailed study of the role of *NEAT1* as a hub for miRNA biogenesis in cardiomyocytes is warranted.

Previous reports regarding the role of miR-22 in regulating apoptosis and the cellular response to hypoxia is rather ambiguous. Whereas miR-22 has been shown to promote apoptosis in a number of cell types including cardiomyocytes (refs)^28,35–41^, there are studies reporting that overexpression of miR-22 inhibits cardiomyocyte apoptosis in rat hypoxia/reperfusion models^42,43^. In the human cardiomyocyte model system used in this study, however, the levels of miR-22 was clearly positively correlated with sensitivity to hypoxia. It is evident that the effects observed by us and others are context- and possibly species-dependent and that more work is needed in order to decisively enunciate the connection between miR-22 and the cardiomyocyte response to hypoxia. According to miRTarBase 2020^44^, there is a total of 39 experimentally validated miR-22-3p target genes. Gene ontology analysis of these genes reveal a significant enrichment of genes involved in “Positive regulation of apoptotic process”, “Regulation of oxidative stress-induced intrinsic apopotic signaling pathway”, “Regulation of muscle cell apoptotic process”, and “Cellular response to hypoxia”. Genes that are common to all these pathways are *HIF1A* and *SIRT1*, which might constitute key miR-22 targets in the regulation of apoptotic pathways in response to hypoxia in cardiomyocytes. Additional studies, including comprehensive mapping of miR-22 target mRNAs through e.g. AGO2 RIP-Seq^45^, are needed to confirm and expand on the exact mechanistic role of the miR-22 targetome in the response of cardiomyocytes to hypoxia.

We acknowledge that the lack of in vivo evidence for the cardioprotective role of *Neat1* is an important limitation of this study, and a factor that must be taken into account when discussing the wider generalization of our results. Du et al.^26^ recently reported that lentiviral knockdown of *Neat1* reduced infarct size in a mouse model of ischemia/reperfusion injury, but the authors did not show evidence for a specific effect on cardiomyocyte *Neat1*. Given that *Neat1* also plays important roles for immune cell^43^ and endothelial functions^44^, a direct role for cardiomyocyte *Neat1* in protecting the myocardium in vivo has yet to be elucidated^46,47^. The fact that *Neat1* is ubiquitously and highly expressed across tissues and cell types means that specific pharmacological knock down of cardiomyocyte *Neat1* via an siRNA or antisense oligonucleotide strategy is unrealistic. As such, cardiomyocyte-specific targeting of *Neat1* via conditional genetic knock out prior to ischemia/reperfusion would be the ideal model to corroborate our findings, and is essential for the implementation of our results in a clinical setting.

## Supplementary information

Supplementary Figure Legends

Supplementary Figure 1

Supplementary Figure 2

Supplementary Figure 3
