# Supplementary material for: Inhibition of the long non-coding RNA NEAT1 protects cardiomyocytes from hypoxia in vitro via decreased pri-miRNA processing
Source: Cell Death Dis. 2020 Aug 13;11(8):677. doi: 10.1038/s41419-020-02854-7 (PMC7442835; doi:10.1038/s41419-020-02854-7)
Supplement: Supplementary file 1 — Supplementary Figure Legends [file 41419_2020_2854_MOESM1_ESM.docx]

Supplementary Figure Legends, Gidlöf et al

Supplementary Figure 1. A) qRT-PCR analysis of *NEAT1* expression in A) HL-1 and B) iPS-CM transfected with scrambled negative control siRNA (siScr) or siRNA to *NEAT1*, normalized to *GAPDH* and expressed relative to the mean of the negative control (HL-1, n=3; iPS-CM, n=9).

Supplementary Figure 2. A) Expression profiling of 96 miRNAs in iPS-CM (n=3) using qRT-PCR. Results are expressed relative to the global mean (dotted line).

Supplementary Figure 3. A) qRT-PCR analysis of miR-22 expression in iPS-CM transfected with unspecific negative control pre-miRNA (pre-miR-Ctrl) or pre-miR-22, normalized to U6 and expressed relative to the mean of the negative control (n=3). B) Quantification of Caspase 3/7 activity in iPS-CM 72 hours after transfection with pre-miR-Ctrl or pre-miR-22 using a luciferase assay (n=3). Mean and standard deviation is shown.
