# Supplementary figures and images for: Inhibition of the long non-coding RNA NEAT1 protects cardiomyocytes from hypoxia in vitro via decreased pri-miRNA processing
Source: Cell Death Dis. 2020 Aug 13;11(8):677. doi: 10.1038/s41419-020-02854-7 (PMC7442835; doi:10.1038/s41419-020-02854-7)

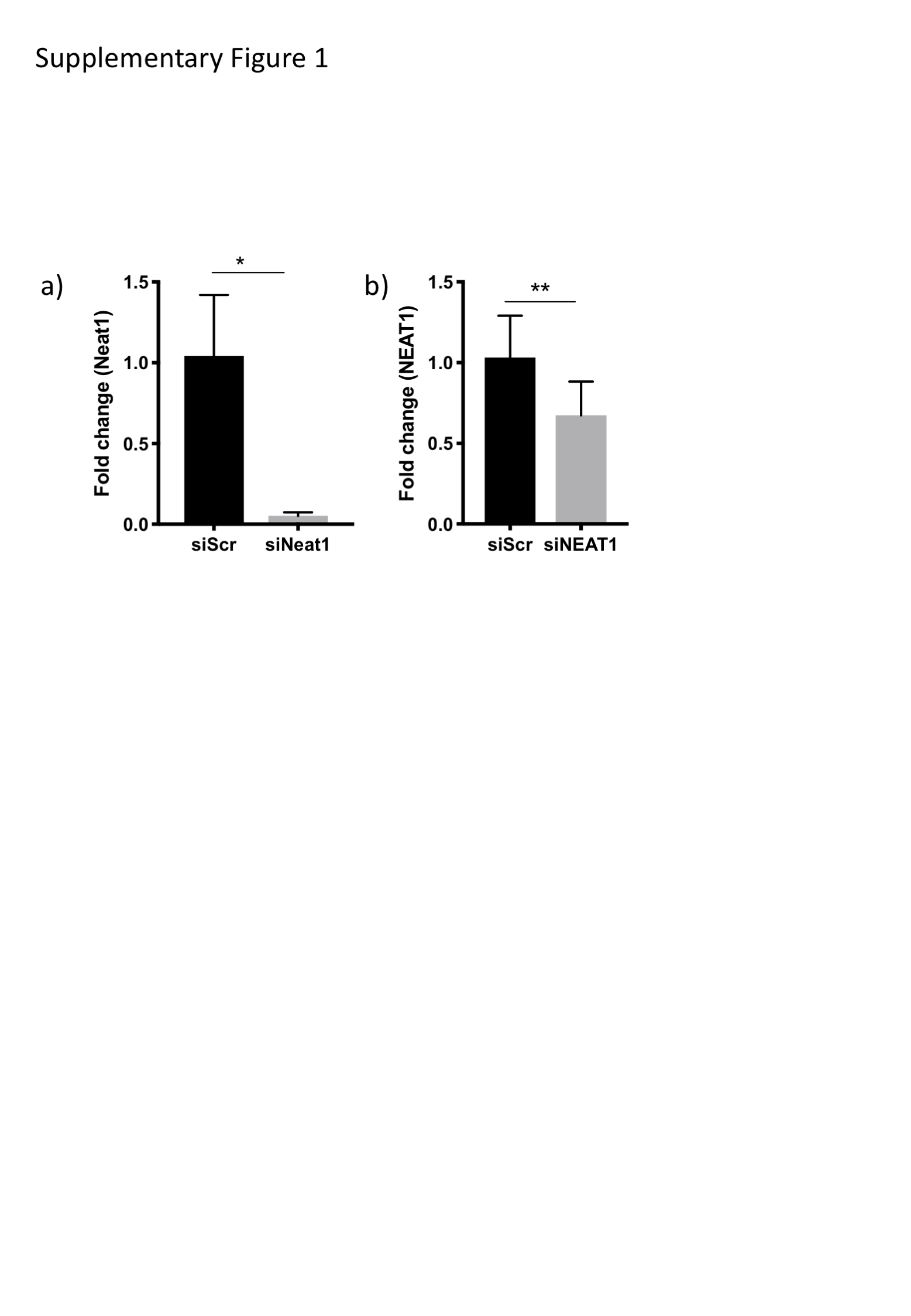

Supplement: Supplementary file 2 — Supplementary Figure 1 [file 41419_2020_2854_MOESM2_ESM.tif]

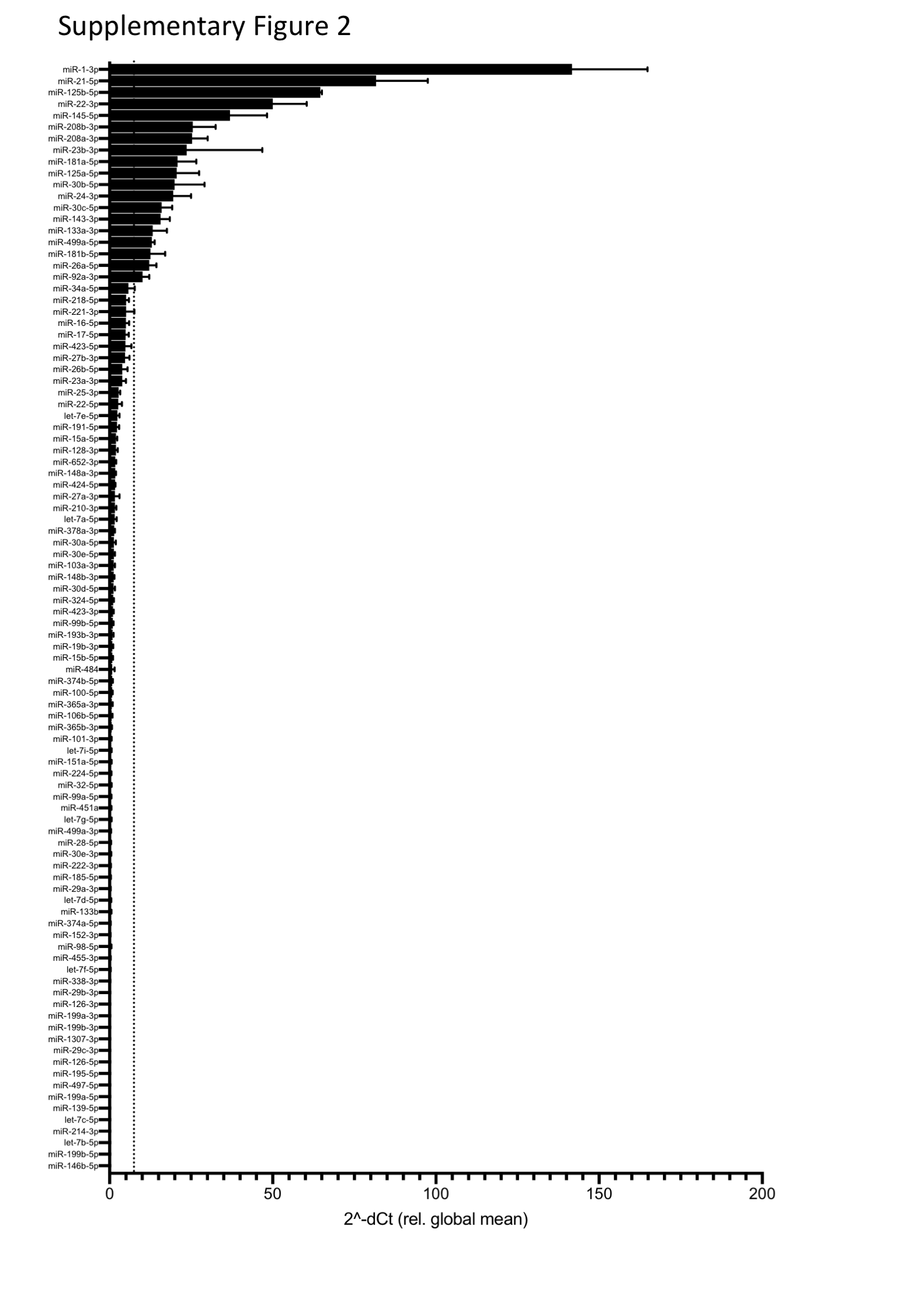

Supplement: Supplementary file 3 — Supplementary Figure 2 [file 41419_2020_2854_MOESM3_ESM.tif]

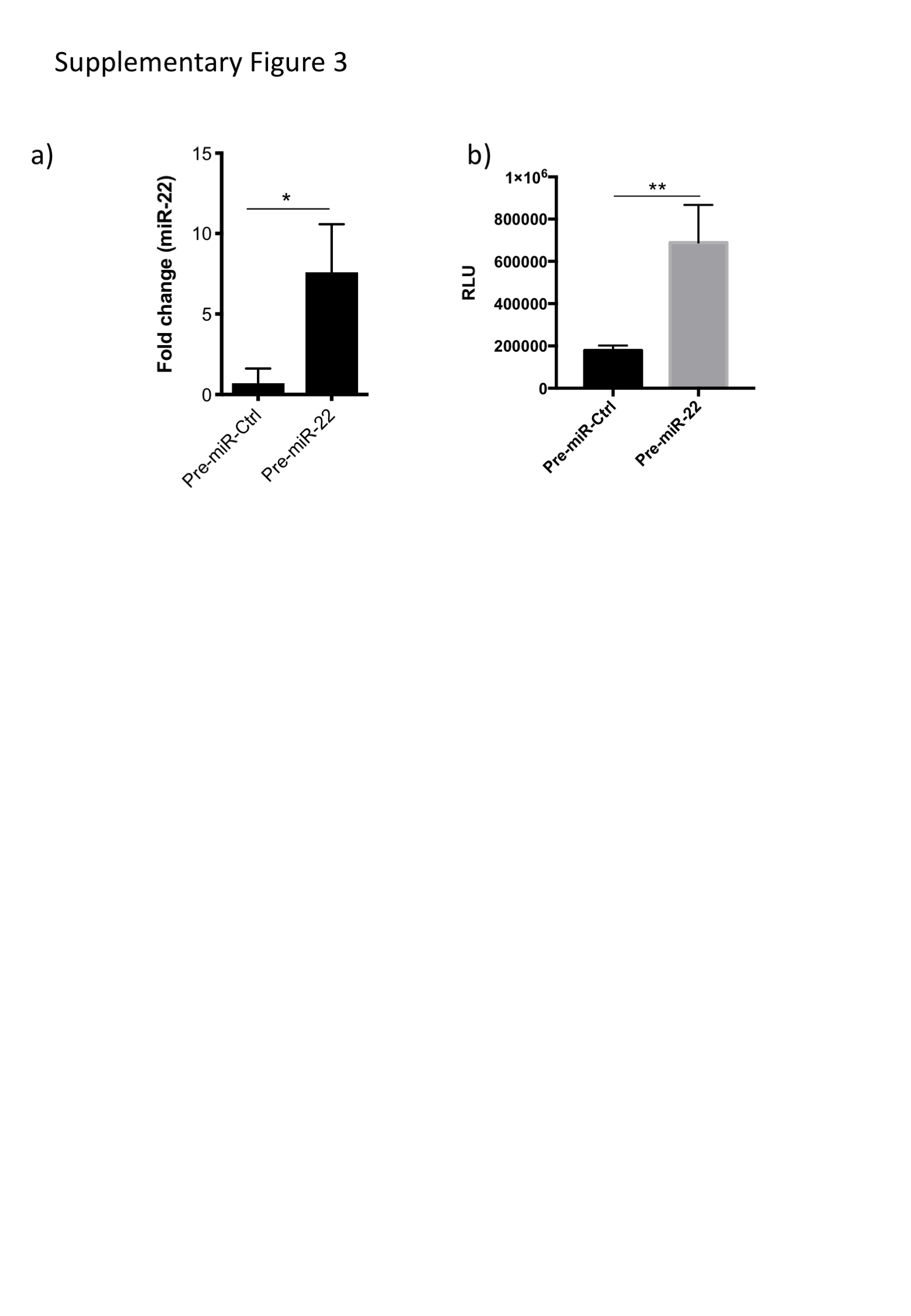

Supplement: Supplementary file 4 — Supplementary Figure 3 [file 41419_2020_2854_MOESM4_ESM.tif]
